# Extraordinary claims, extraordinary evidence? A discussion

**DOI:** 10.3758/s13420-021-00474-5

**Published:** 2021-08-10

**Authors:** Richard M. Shiffrin, Dora Matzke, Jonathon D. Crystal, E.-J. Wagenmakers, Suyog H. Chandramouli, Joachim Vandekerckhove, Marco Zorzi, Richard D. Morey, Mary C. Murphy

**Affiliations:** 1 Indiana University, Bloomington, IN USA; 2 Department of Psychological Methods, Room G 0.29, University of Amsterdam, Nieuwe Achtergracht 129B, PO Box 15906, 1001 NK, Valckenierstraat 59, 1018 XE Amsterdam, the Netherlands; 3 University of Helsinki, Helsinki, Finland; 4 University of California, Irvine, Irvine, CA USA; 5 University of Padova, Padova, Italy; 6 IRCCS San Camillo Hospital, Lido Venice, Italy; 7 Cardiff University, Cardiff, UK

**Keywords:** Evidence, Publication criteria, Numerical cognition, Extraordinary claims, Comparative cognition

## Abstract

Roberts (2020, *Learning & Behavior, 48*[2], 191–192) discussed research claiming honeybees can do arithmetic. Some readers of this research might regard such claims as unlikely. The present authors used this example as a basis for a debate on the criterion that ought to be used for publication of results or conclusions that could be viewed as unlikely by a significant number of readers, editors, or reviewers.

## Rich Shiffrin

When I saw a report by Roberts (2020) summarizing research showing that honeybees can do arithmetic, I was intrigued by the claim: As a nonexpert in fields of animal cognition and models of numerosity, my initial reaction was skepticism: Especially when I think of the difficulty of teaching mathematics in secondary education in the U.S., the claim that honeybees do arithmetic of the sort we attempt to teach students seemed unlikely. As we shall see in the dialogue to follow, there are decent arguments that honeybees could produce the data in the experiments in question. Thus, the judgment that this claim is unlikely might apply more to laypersons and scientists not in the fields that are most relevant. Regardless of the validity of the claim, my initial reaction led me to think about an issue that has come up in various forms in discussions about the “reproducibility crisis”: What should be the general criterion for publication, especially when a submission contains findings or conclusions that some editors and reviewers, and many laypersons, might find unusual or unlikely? Should such reports be published by the same somewhat lenient criterion used for expected results and conclusions? One argument for doing so would be the hope that validity of the report will be pursued in further research. Alternatively, should exceptional measures with exceptional strong degrees of support be demanded before publication of such research? Carl Sagan is reported to have said “extraordinary claims require extraordinary evidence.” My interpretation of this statement is not that extraordinary claims should require extraordinary evidence for a first publication, but rather that for an extraordinary claim to be established and accepted as the current best account, extraordinary evidence is needed. However, general acceptance of an extraordinary claim usually takes place at the end of a lengthy series of investigations and tests. Thus, I lean toward leniency for initial publication of extraordinary and unlikely claims. In my view a strict criterion at the outset of the publication process would stifle progress, producing a literature that reifies what is presently thought to be known. I am willing to accept many false leads and results that cannot be reproduced so that a few new and important results can be uncovered. The research on arithmetic by honeybees may or may not be judged as unusual or unlikely, especially by domain experts, but the issue of criteria for publication is an important one and is the subject of the present dialogue.

## E.-J. Wagenmakers

I agree that it is unwise to suppress data or ideas. However—and this is highly unfortunate—in order to qualify for publication, a paper generally requires a strong claim. When these claims are extraordinary, many of them will prove false; publishing such false claims on a regular basis pollutes the literature and erodes trust. Researchers will find themselves chasing gaggles of wild geese, instead of just one or two. Moreover, once published, a claim is almost impossible to dislodge from the literature. For example, the infamous study on the relation between autism and vaccination (Wakefield et al., 1998) has had a profound negative real-life impact, even after the study was thoroughly discredited and even retracted.

## Jonathon Crystal

To provide some context, Roberts (2020) wrote an *Outlook* paper in *Learning & Behavior* focused on a recent paper by Howard et al. (2019) in *Science Advances* (and an earlier study by the same group [Howard et al., 2018] in *Science*; see also Cordes, 2019). Briefly, Howard et al. (2019) reported that bees were presented with an initial pattern of yellow geometrical shapes and then were confronted with two additional yellow patterns; one choice pattern had one more element than the initial pattern (+1 training) and the other had a different number of elements. The +1 choice was rewarded with sucrose, whereas the other choice was punished with bitter quinine. On other occasions, the patterns were blue and the choice was between a pattern that had one less element than the initial pattern (−1 training). The bees learned the discrimination, but this can be solved by memorizing the example cases without any numerical competency. To test for addition and subtraction, the experiment continued by using an initial pattern with a number of elements that had *not* been presented earlier in training. The bees generalized their earlier learning about the rule (+1 or −1) using a novel number of items in the initial pattern; the choice patterns had numbers that the bees had experienced in training. Studies of apparently complex cognition in an invertebrate (with a brain the size of a sesame seed) may suggest that a precursor of this aspect of cognition was present in a distant species hundreds of millions of years ago or that this aspect of cognition is much simpler (in terms of neural computations) than previously supposed. As a further bit of context, bees are renowned for surprisingly complex behavior (von Frisch, 1967), including recent claims that bees may have precursors of episodic memory (Pahl et al., 2007) and metacognition (C. J. Perry & Barron, 2013).

Whether an unexpected cognitive feat in insects is viewed as an example of an extraordinary claim may be judged relative to the numerous demonstrations by different research groups, using different species, and different cognitive tasks. Some examples may help to set the context. With respect to numerical cognition, bees have been widely reported to show sensitivity to number (Chittka & Geiger, 1995; Dacke & Srinivasan, 2008; Gross et al., 2009; Howard et al., 2018). More broadly, examples of apparent cognitive feats in bees include categorization of face-like stimuli (Avarguès-Weber et al., 2010), using multiple abstract concepts (Avarguès-Weber et al., 2012), judgements of same and different (Giurfa et al., 2001), cognitive flexibility (Loukola et al., 2017), and cross-modal object recognition (James, 2020; Solvi et al., 2020). Across the animal kingdom, quantitative representations of quantity are pervasive (Brannon, 2006), although, admittedly, not typically demonstrated by addition and subtraction.

## Suyog Chandramouli

The points made by Rich and E.-J. concern the influence of publication criteria on the progress of science. Definitive answers to questions about scientific progress are hard to obtain due to difficulties in (i) conducting controlled experiments to test the effect of interventions in the publication process, and in (ii) quantifying scientific progress (Cowen & Southwood, 2019; Shiffrin et al., 2018). Nevertheless, many paradigm shifts in science have been driven by claims that had once seemed extraordinary (Kuhn, 1962), so suppressing extraordinary claims could, at worst, enable confirmation bias where only data deemed plausible by popular theories are published, holding back step changes in science. In contrast, the “reproducibility crisis” (Baker, 2016; Open Science Collaboration, 2015; Pashler & Wagenmakers, 2012) has highlighted how increasing numbers of extraordinary, invalid, irreplicable, and hyped claims could hinder science. This leaves reviewers and editors with a quandary when faced with seemingly extraordinary claims that, if explored, could push their fields forward.

There are many potential solutions to this quandary. A simple solution is to append to publications reviewers’ thoughts on the plausibility of the purported claims based on current theories and their actual reasons for accepting submissions. Similarly, one may want to allow for curated postpublication peer reviews (Knoepfer, 2015) and opinions about the claims by a diverse range of experts. Such reviews, if highlighted, can go a long way in preventing hype and moderating beliefs about preliminary reports without hurting potential progress. With the help of versioning, the original authors could also update and improve upon the original articles. Explorable multiverse analyses (Dragicevic et al., 2019; Steegen et al., 2016) can allow the reader to explore plausible forking paths in statistical analyses and make up her own mind about the robustness of a claim. Linking to the original report any follow-up attempts towards replicating or extending the claims could be yet another way to enable the reader to assess the validity of the claims over time.

## Richard Shiffrin

I believe the dangers of publishing unlikely results without correspondingly strong evidence are exaggerated. Most such publications are simply ignored, as are roughly half of all publications. The relatively few judged to be important are followed up, and this is not a waste of time but a way to advance science, whatever the eventual result. Of course, there are exceptions, such as reports that are important for immediate benefit or harm to society, but such cases should not be used to formulate a general rule. Judging which results are worth following up is and should be a core part of the practice of science. A publication is not a single event but one element in an extremely long chain of experimentation and theory development.

## E.-J. Wagenmakers

**Fig. 1:**
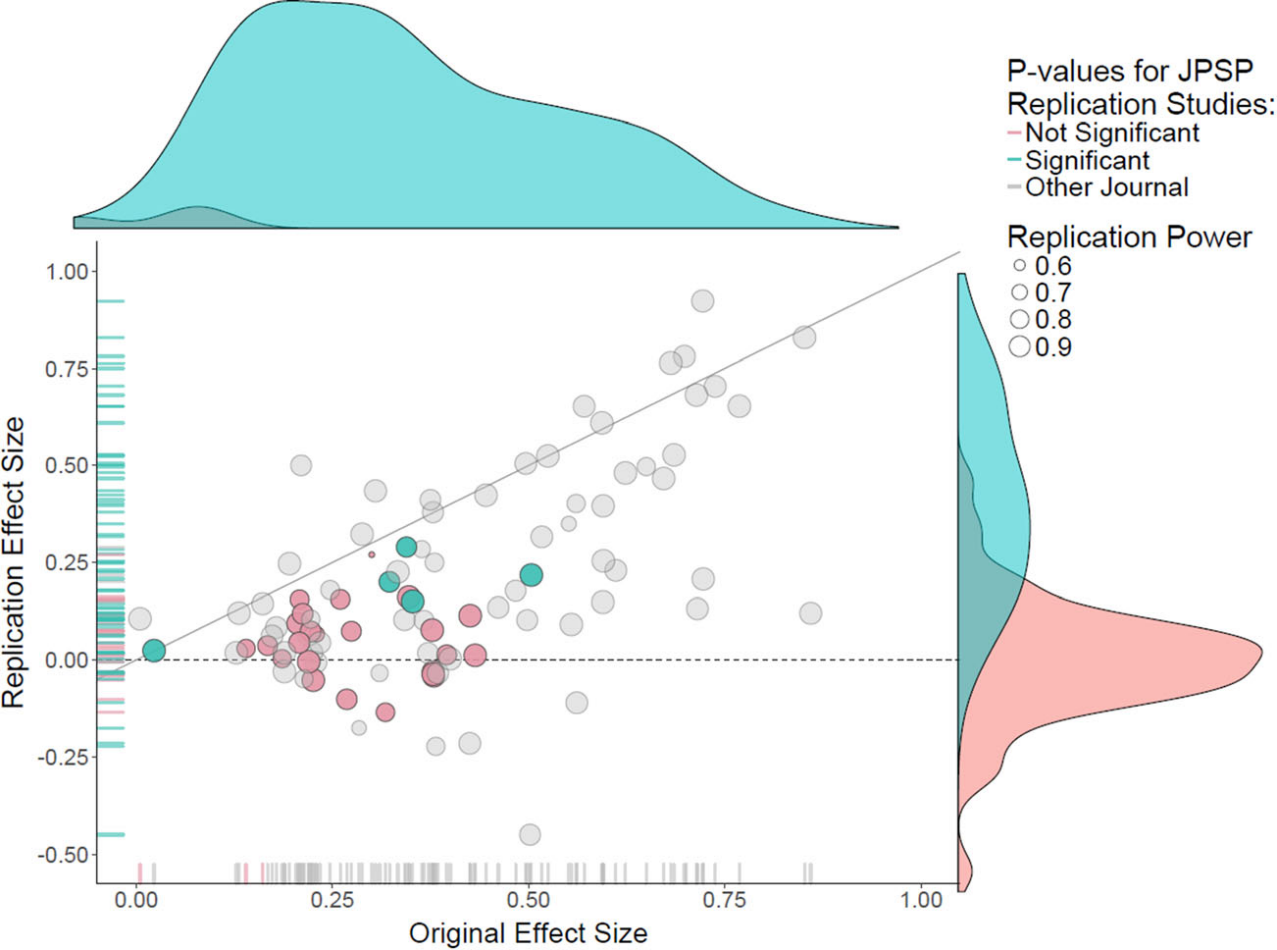
Results from the replication attempts conducted as part of the *Replication Project: Psychology*. The results for the replication attempts of JPSP articles are highlighted. Courtesy of Fred Hasselman, this graph is an edited version of the one presented in Open Science Collaboration (2015). The associated information is available on the Open Science Framework (https://osf.io/ezcuj/). Figure and associated text from Wagenmakers et al. (2017)

I fear that, once untethered by the requirement to provide strong evidence for implausible claims, a literature may become so polluted with false findings that it will make it very challenging to decide which results are worth following up and which are not. As a first example, consider the literature on behavioral priming, the idea that people’s behavior is influenced by the unconscious impact of subtle conceptual cues that were provided minutes or even months earlier (e.g., Kahneman, 2011). This entire literature has proven spurious—the empirical studies do not replicate. As a second example, consider the semirandom collection of studies from the flagship journal of social psychology, *Journal of Personality and Social Psychology* (JPSP), that were subject to a large-scale replication effort (Open Science Collaboration, 2015). Figure 1 shows the result with the JPSP studies highlighted. As concluded in Open Science Collaboration (2015), “average effect size in JPSP did not differ from 0 (.042; *z* = .71, *p* = .48)” (p. 45). The left-most green dot reflects a significant effect because it was a psychometric study with 230,047 participants. As mentioned in Wagenmakers et al. (2017):

Figure 1 paints a bleak picture: all but one of the replicated effect sizes lie below the diagonal, meaning that they are smaller than those originally published in JPSP; some replicated effect sizes are negative, whereas others are so small that—even if these effects were real—it would take much larger sample sizes for them to be meaningfully studied (e.g., Button et al., 2013); finally, 6 out of 31 replication studies were statistically significant, compared to 30 out of 31 for the original experiments published in JPSP.

These examples lead me to believe that there is substantial value to a scientific literature in which it is clear which results are trustworthy and which are not.

## Jonathon Crystal

Replication in research using animals has received increasing attention (Beran, 2020; Farrar et al., 2020; Vonk & Krause, 2018). It is worth noting that a fundamental ethical principle in research using animals is to minimize the number of animals used in research (Russell & Burch, 1959). Some degree of replication is warranted, but each study is required to minimize the number of animals and is proscribed from unnecessarily repeating experiments. A further constraint on widespread replication efforts is the limited availability of some animals (e.g., nonhuman primates). Consortia offer a potential avenue for pooling resources and achieving some degree of standardization (e.g., Altschul et al., 2019).

## Joachim Vandekerckhove

The claim that publications with unlikely results go ignored is not in evidence. It appears that many publications do not have follow-up papers, but this is equally consistent with many failed and costly replication attempts whose results have been relegated to file drawers.

Ultimately, we do want there to be a demarcation criterion between *this* literature, which can be trusted and safely consumed, and *that*, which cannot. While it may not be a sufficient criterion, I would inelegantly argue that a necessary criterion is that *the results described in published papers should not be extreme outliers relative to the population of outcomes of the empirical procedures described*. That is to say, results described in a paper should describe the outcome of the study that was described, and should not be subject to additional post hoc selection. If publications are filtered by their results, this condition is violated, the published results are no longer an unbiased realization of the empirical procedure, and such publications therefore belong on the untrustworthy side of the line (see, e.g., Sterling, 1959).

A reviewer pointed out that by this criterion, the bee papers are not outliers. The reviewer may be right, but as a consumer of this literature I have no tangible way of confirming whether this is true or not, since I do not have access to the complete population of germane bee studies that were actually performed—as a consumer, I see only the published sample. There are presumably many ways of determining whether a literature is subject to censoring—public preregistration of data collection is one of them. (Another method that comes to mind is the reasonably common practice of sending surveys to large groups of researchers in a particular field in the hopes of cataloging their unpublished studies. This, of course, relies on those researchers being generous with their time, forthcoming with their unpublished data, reachable, and, not to put too fine a point on it, still alive.) Various statistical remedies for publication bias at this time do not appear to be effective (Carter et al., 2019). On balance, I believe public preregistration of data collection to be the most viable approach to avoiding publication bias—whether the bias is towards papers that report conclusive results, confirmation of a cherished theory, or merely towards strong evidence.

## Rich Shiffrin

Joachim’s comments might be sensible if doing science was a matter of doing statistics. The results that move science forward are often those that are “*extreme outliers relative to the population of outcomes of the empirical procedures described*” because no studies are exact replications of others, and small differences in procedures are often the key to unlocking new discoveries. We should be lenient in publishing unlikely results, despite knowing that discriminating the new findings that move science forward from the many that are invalid and/or irrelevant is difficult—that is where good scientific judgment comes into play.

## Joachim Vandekerckhove

To clarify (and argue for sensibility), regardless of whether a study is a replication or a new, unique design, if a researcher decides to publish their results based on some data-dependent criterion (like only large effect sizes or strong evidence), then the data they report can no longer be interpreted as resulting from the study they conducted. This is independent of whether the results are unlikely in some prior sense; the data reported are simply not representative of the outcomes that might be expected from the study. It is not clear to me what the data are good for at that point.

## Mary Murphy

This entire conversation strikes me as one about gatekeeping that has been central to conversations about replication and reproducibility. Rich asks, What should be the evidentiary criterion for publishing unusual findings? And Joachim asks, What is the criterion for literature that can be trusted versus that which cannot? It seems to me that we should question the questions here. Can’t there be multiple criteria that would make an article and literature valuable contributions? Does replicability always have to be one of those criteria? Surely there is value in some nonrepeatable science (for example, investigations with rare populations or of infrequent events).

I am with Rich in that I view science as a process involving the accumulation of many tests and investigations—each with varying degrees of what, by today’s standards, constitute best practice (e.g., specified theory and connection to data, preregistration, open materials, open data, open code). In my view, well-powered, methodologically and contextually sound studies that produce either unusual/extraordinary results or that produce null results convey important information that could shape future theory and research—and neither of these results should be suppressed. At the same time, it seems that labeling literatures and articles trustworthy or untrustworthy invites unnecessary judgment that may, over time, prove itself inaccurate as tools, methods, theories, and hypotheses develop. For “open science,” it feels quite ironic to put up relatively rigid, narrow criteria by which we judge scientific contributions as worthy, especially since those criteria are likely to shift over time.

## Rich Shiffrin

E.-J. discusses Kahneman’s conclusions about priming effects, and the fact that many of the claims in the literature cannot be replicated. Yet priming generally is one of the best established and strongest effects in the cognitive and psychological sciences, and has both extremely strong empirical evidence and excellent theory to back it up (Huber et al., 2001). Of course, the size of priming effects varies widely across contexts, and one can expect some would be too small to measure with reasonable-sized studies, especially those that seem unlikely on the face of things. The question of the harm done by publication of unlikely priming effects that are too small to be measured, and conclusions drawn from them, is a subtle one. There are two elements of harm that are particularly salient: (1) The field of psychological and cognitive science is called into disrepute by demonstrations that the effects, if any, are too small to measure. (2) The examples of priming failure call priming effects generally into question, despite overwhelming evidence of their importance. Both these examples of harm are produced by those who have decided to pursue the unlikely examples of priming and show they are too small to measure. Without those demonstrations, I think the harm done to science would be modest, though not negligible—for example, leading students to pursue priming effects that are unlikely and “wasting” their efforts. I would hope that good mentors would point out that such effects, if true, would be hard to measure, and point their trainees toward effects more likely to be fruitful avenues for investigation.

## Jonathon Crystal

Replication-crisis challenges also include basic learning mechanisms. Blocking and overshadowing are core findings in associative learning (Kamin, 1969), which has played a fundamental role in theory development. Nonetheless, a recent paper (Maes et al., 2016) reported 15 cases of failures to replicate blocking.

## Suyog Chandramouli

I believe that if consumers of science were taught to be more skeptical of empirical findings that are unexpected per prevailing theories, then their negative impact when they don’t eventually pan out would be reduced. This is better than disallowing papers based solely on the magnitude of the reported effect size, which would be a form of publication bias; publishing unlikely claims, perhaps with disclaimers from reviewers can, on the other hand, still allow interested researchers to follow promising leads.

I agree with Rich that results from replication studies should be interpreted carefully. Ideally, replication attempts provide information about the empirical robustness of the individual studies in question. They may be useful to carry out when some findings have important social and scientific implications, when there are few resource constraints in conducting them, or when the targeted effects are those that are taken for granted in the literature with neither formal theoretical backing nor an adequate amount of testing.

In the context of priming, where there is also strong evidence favoring it in certain contexts, one may not want to infer from small effect sizes in replication studies that priming effects do not exist in general. A better approach would be to jointly use known credible findings along with replication data to build a better theory about the factors that influence the salience of priming effects. Such a theory could then be tested with an experiment that attempts to maximize informative effects while minimizing noise in the design.

## E.-J. Wagenmakers

Maybe I should regret having brought up behavioral priming. The intent was to demonstrate how an entire subdiscipline can go astray and for decades remain stuck in a self-perpetuating cycle of producing noise masquerading as signal. I should stress that semantic or orthographic priming is a far cry from the kind of behavioral priming that is at stake here. After explaining that people are reliably faster to recognize and respond to the word “cat” after having been exposed to the word “dog” a few seconds earlier (i.e., semantic priming), David Meyer (2014) draws a vivid contrast to behavioral priming: “Viewed from a metaphorical perspective, what some social psychologists have done is essentially like trying to show that presenting the printed word ‘dog’ may incline English-reading adult male humans more toward visiting remote ‘cathouses’ (slang for brothels) even after substantial amounts of time (several minutes or more) have elapsed since the original exposure to ‘dog.’ Much further research is needed for assessing to what extent such behavior priming effects are real.” p. 523

I do not believe there is much progress to be made by assuming that these “cathouse” effects are small and real instead of completely spurious. And modeling is laudable and potentially insightful, but not when the data are almost 100% noise—it would be like taking a Ferrari and driving it straight into a swamp. More to the point, although I am in firm agreement that “results from replication studies should be interpreted carefully,” it seems to me that results from original studies should be interpreted even more carefully, and particularly so when the claim is extraordinary.

There may be occasional value to the publication of an extraordinary claim even in the absence of extraordinary evidence, but perhaps journals ought to create a special section for this kind of work (i.e., “Speculation,” or just “Extraordinary Claims”).

## Marco Zorzi

One major shortcoming of the “extraordinary claims require extraordinary evidence” approach stems from the subjectivity of the judgment. What looks extraordinary to you might be less surprising to me: One’s judgment is heavily weighted by prior knowledge and biases. This was exactly the case for the article that spurred the present discussion. In particular, I was predisposed to accept the finding that honeybees can do arithmetic—thereby judging it as very interesting but nonextraordinary—based on previous theoretical work showing that basic number skills can emerge even in quasirandomly wired neural networks (Zorzi & Testolin, 2018; also see Hannagan et al., 2018). How can we decide on whether a paper should be relegated to a special “Speculation” section of the journal, as proposed by E.-J.? Shall we ask each reviewer to rate how much the claims are extraordinary on a 5-point scale or provide a (posterior) probability? We would then also need to identify a suitable cutoff for the special section, and perhaps even one for outright rejection based on the extraordinary criterion. Overall, I see more harm than good in this approach, because it resonates with the complementary problem of weighting editorial decisions more on perceived impact (e.g., *Nature*’s criterion of “outstanding scientific importance”) than on the more objective judgments about the quality of the work.

## Mary Murphy

Extraordinary claims can only be labeled extraordinary relative to ordinary claims. Without ordinary claims, there is no such thing as extraordinary claims. Ordinary claims are rendered ordinary because they are consistent with prevailing theory, paradigms, methods, and results. And much of this prevailing knowledge is related to who has been conducting science and how they have been conducting science across time. If we apply stringent criteria for what “counts” as extraordinary, we are limiting our science to that same prevailing knowledge and the same types of scientists who have historically produced that work. The makers of such criteria should come prepared with ideas for how these consequences will be addressed and remedied.

## Jonathon Crystal

The observation of numerical competencies in bees may be seen as surprising for what it says about bees (more complex than expected), or for what it says about human cognition (less complex than expected). The same dichotomy of inferences applies to surprising findings in animals more generally. I’ll offer a personal example of straddling this division. Some years ago, I published a paper claiming that we had documented metacognition in rats (Foote & Crystal, 2007; see also Kepecs et al., 2008; Templer et al., 2017). We had adapted a method that was considered the gold standard in monkey research (Hampton, 2001). At the time, I believed the evidence supported metacognition in rats. Our data led a group of primate metacognition researchers to develop a simple model that captured key aspects of our data with rats and data from monkeys, but the model did not include metacognition (Smith et al., 2008). My reaction (after simulating the model and observing the same pattern as in our data) was to change my view. I no longer believe that rats demonstrate metacognition (Crystal, 2012, 2014, 2019; Crystal & Foote, 2009a, 2009b, 2011). A number of simple models also suggest that monkeys and rats do not demonstrate metacognition (Crystal & Foote, 2009a; Jozefowiez et al., 2009; Le Pelley, 2012, 2014). Perhaps the headline that rats demonstrate metacognition was a misdirection, but in this instance it changed what we think about metacognition in nonhuman primates.

## Marco Zorzi

Jonathon’s case about metacognition in rats is very interesting because it suggests that the empirical findings connected to the (putative) extraordinary claim might hold up in replication, so the claim can be rejected only by offering a more parsimonious (and probably less extraordinary) theoretical account. The latter “extraordinary claim scenario” is very different from the one discussed earlier in connection to replication failures: Improving data collection and statistical inference methods would not help here. Nevertheless, the theory level is likely to play an important role in the replication crisis. Indeed, weak connection between theory and empirical testing has been highlighted as a further cause of poor replicability (see Oberauer & Lewandowsky, 2019, for a thorough discussion). The metacognition case also highlights (if at all necessary) the virtue of formalizing theories into computational models and using simulations to connect theory and data (at least in a “theory-testing” scenario; Oberauer & Lewandowsky, 2019). This has been the standard approach for more than 30 years in the field of visual word recognition and reading aloud (see C. Perry et al., 2007, for discussion). An innumerable number of published studies have contrasted hypotheses derived from different computational models of reading, thereby assessing their empirical findings against models’ predictions. In contrast, extraordinary claims (and theories) have proliferated in the close field of dyslexia, and the use of computational modeling has been more the exception than the rule. Again, modeling might be a game changer when it is used to formally compare different theories of dyslexia (C. Perry et al., 2019).

## Rich Shiffrin

The importance of theory cannot be overestimated, but the implications for publication criteria are complex. Daryl Bem became notorious for publication of two articles in high-quality journals claiming the existence of ESP (Bem, 2011; Bem & Honorton, 1994). The experimental design and the statistical power looked persuasive enough to lead the editors and reviewers to a decision to publish despite the lack of a theory to explain the results. Should the lack of a theory have been used to suppress publication? I think not. After all, physicists have been led to propose “dark matter” and “dark energy” despite lack of an accepted theory (albeit a variety of theories have been proposed; e.g., Kastner & Kauffman, 2018). In addition, publication of results and claims without a good theory could lead to theory in the future, or could lead to investigations showing the pervasiveness of experimenter bias and error in science. At the other extreme, publishing unusual results with a new theoretical account is often the way science progresses, when further research shows the validity of the new theory (e.g., the acceptance of quantum mechanics; e.g., Müller-Kirsten, 2006).

## Richard Morey

If the literature can be considered the public part of scientific practice, as many of us would like it to be, then we should—must, really—trust other scientists to reason with results as well as we do. To do otherwise would disrespect their agency and raise the potential for deception. We will experience many scientific blind alleys in our own work; to the extent that these inform our own conclusions, they can and should also inform others. They are both a necessary aspect of scientific progress (not something to be avoided!) and often unclear when they occur, maybe even for years. The problem is, and has always been, (1) selective publication, and (2) that the anything in the *literature* is trusted too much (and selectively).

Having failed in my PhD to replicate a rather famous affective priming effect, and then being told by a prominent priming researcher that everyone knew that the paradigm doesn’t “work,” I definitely feel the tension. I think research communities often have this knowledge without a way to efficiently use it.

What we lack, for historical and technological reasons, is a tiered approach to publication that mirrors the way we think about evidence. Experiments that have no business being taken as strong evidence for broad claims end up being so because publication is an either–or proposition. A system that separates, to a larger extent, publication of results from publication of claims, and that allows a more nuanced view of the quality of evidence, would be welcome. Methods and basic results for all experiments we run, along with methodological post mortems, should be published as tech reports (perhaps in appropriate official outlets). This would allow the “everyday science” to be seen by everyone, warts and all. Scientific progress occurs through synthesis of results, and at this level is where scientifically interesting claims should happen (I note that this is how people think of the journal *Psychological Review*: a place for synthesis).

I tend to think that science being more public all around (including preregistration of experimental methods, which are then augmented with results) would prevent much of the nonsense we see. But to reiterate: science is not straightforward, and people will be led astray, potentially for decades, sometimes. This is something that has to be allowed and even celebrated as a core element of the scientific process. We should be allowed to fail, in good faith.

## Rich Shiffrin

An issue raised by a reviewer concerns differences in policies among journals. Because the number of journals in our fields (as well as others) has proliferated in recent years, because prior reviews and editorial decisions are not always transferred when a rejected article is submitted elsewhere, and because there is always noise in reviewing and editorial decisions, an article rejected from one or more journals can be submitted to others until an acceptance occurs. This present state of affairs could be an argument for a strict publication criteria if used by all or most journals. This is not the case at present. Even if all journals would agree to use similar criteria for publication, the present dialogue shows disagreement concerning what that criterion should be.

A related issue raised by a reviewer concerns the editor’s choice of reviewers. How this factor should affect choice of publication criteria is not obvious. If reviewers are chosen to be knowledgeable and sympathetic to the claims, that would in effect produce a liberal criterion, but if knowledgeable and skeptical of the claims, it would in effect produce a strict criteria.

## Dora Matzke

Following up on Richard Morey’s suggestion for a tiered publication approach, I think it is important to acknowledge the role of the present publication culture and the academic incentive system in this discussion. I agree with previous comments that science should be built on cumulative knowledge, acquired over multiple studies of varying evidential value. I believe that studies producing “ordinary” results can be important for scientific progress, and there is no way of knowing in advance if an ordinary result supporting an extraordinary claim is just a fluke or the basis of the next groundbreaking theory. If a study is methodically rigorous and the data are theoretically well-situated and openly available, I think the study deserves publication.

However, I also think there are some efficiencies to be gained: for psychology as a field, it took Bem’s (2011) extraordinary claim that people can look into the future to trigger a large-scale reevaluation that ended up rejecting various claims that had slipped into the literature and even in our undergraduate textbooks as facts. I do not think that suppressing scientific claims based on subjective judgments about the trustworthiness or the degree of extraordinariness is the solution, but I believe that as a scientific discipline we should exercise some more humility and do our best to acknowledge the uncertainty of our results, claims, and even our standards and best practices.

However, this is easier said than done. We do not do science in a vacuum: We want others to read and build on our work, and we need publications to climb the academic ladder. Consequently, we must view the question as to how we should evaluate the trustworthiness of scientific claims in light of the culture that journals, funders, institutes, and scientists themselves have built. I tend to think that science as it stands is ultimately self-correcting, but that self-correction is often delayed by the current academic structures. In particular, the tendency of journals to reject null results and replication failures contributes to a climate where results that contradict the established scientific fashion are difficult to challenge. Journals also prefer strong claims and clean stories as opposed to the messy reality, and my impression is that the higher impact the journal, the cleaner the stories and the stronger the claims need to be. Current incentives are shaped by funding agencies and institutional tenure committees’ emphasis on the number of publications, impact factors, and citation metrics. This makes it difficult to resist the temptation to take shortcuts, exaggerate claims, and aim for high-impact journals that sometimes place more emphasis on perceived novelty than scientific rigor.

I think that before we can rely on efficient self-correction, where scientific dead-ends reflect the healthy progression of science instead of unhealthy stagnation, we need to change the publishing culture and the incentive system in academia. A tiered publication structure may nicely fit in such a new world, although issues such as how to avoid overburdening peer reviewers, and how to search and summarize burgeoning outputs, will need to be faced.

## Richard Shiffrin

I think that the historical emphasis upon null hypothesis testing has misled many scientists who should know better (especially scientists in the fields of psychological, neural, and cognitive sciences) to view results as “true” or “false.” Consider E.-J.’s statement: “I do not believe there is much progress to be made by assuming that these ‘cathouse’ effects are small and real instead of completely spurious.” Pretty much all effects we study and report are highly context dependent, and all hypotheses, models, and theories based on them are (usually crude) approximations to reality. This implies that scientists need always to exercise a good deal of skepticism concerning reports, whatever is their judged likelihood of the validity and strength of the findings and conclusions, and implies that the exercise of good scientific judgment is a critical component of the practice of science. This would be the case even if a much stricter criterion for publication would be imposed. Of course, a scientist’s job would be far easier if a scientist could rely on reported findings and conclusions drawn from them. However, a system of scientific practice that attempts to implement such a state of affairs would instead produce an enormous bias to reify what is already assumed to be true, and stifle progress.

## Mary Murphy

This discussion of publication criteria for extraordinary claims should not omit discussion of biased criteria applied to the claims (extraordinary or otherwise) of scientists from underrepresented groups. For example, there is evidence that women’s scientific claims are met with more suspicion, including more hostile and patronizing questions by colleagues (e.g., Dupas et al., 2021). If citation rates are an indicator of how scientists value the claims of their peers, global gender disparities disadvantaging women’s claims have been documented (Larivière et al., 2013). Even when the content of scientists’ claims is held constant, as in randomized controlled studies where only the scientist’s gender is manipulated, abstracts by “men” are judged to be of higher quality than those of “women” (Ford et al., 2019). Of course, there are even gender biases about whether research revealing gender bias is perceived as legitimate—or extraordinary, if you will. Men evaluate the quality of research unveiling gender bias as less meritorious than do women (Handley et al., 2015). Finally, the “watchdog effect” suggests that people scrutinize the claims of underrepresented individuals more closely than those of majority groups. That is, people are more likely to question the claims of people of color while giving White people a pass on bad arguments and evidence (Johnson et al., 2017; Petty et al., 1999). Of course, these biases about whose claims are judged worthy have significant career consequences that widen already wide inequalities in science. A recent study suggests that early career scientists from demographically underrepresented groups contribute more novel and innovative scientific discoveries to the literature, but these contributions are taken up by the scientific community at lower rates than similarly novel contributions by gender and racial majorities; indeed, underrepresented scientists’ more innovative contributions are less likely to result in successful scientific careers (Hofstra et al., 2020).

These biases are just one of the reasons why I am skeptical of most predetermined criteria for judging the worthiness of scientific contributions. Women, people of color, and especially women of color know that . . . in the words of Chris Rock: “That train is never late.” I have known too many women of color who have opted out of science because of the gatekeeping and labeling that has become associated with the replication crisis. The biases about the scientific worthiness of claims I describe above are cultural, systemic problems that often apply regardless of one’s own particular identities because they are rooted in sociocultural and historical stereotypes and representations of science. So, the solution cannot be a simple one, such as asking underrepresented individuals to do more reviewing and editing (not to mention the inequitable labor this would require of underrepresented scholars); the solutions should, at least in part, involve questioning the use of criteria itself.

## Richard Shiffrin

Mary makes a strong case that the criterion for publication is subject to a gender bias. Is there a remedy? To a certain extent such biases on the part of reviewers and editors can be reduced by deidentifying authors (though this is hard to do effectively). However, it would not be sensible or appropriate to deidentify the authors when a publication appears. Women authors publishing unlikely data or claims would likely find reactions more negative and skeptical, and would likely encounter a higher probability of subsequent publications attacking their findings and views, than would be the case for male authors. In addition, knowledge or fear that this would occur might make women scientists less likely to submit such manuscripts. Finally, I believe that use of a severe criterion for publishing unlikely data or claims would exacerbate gender biases: Making delicate and fine discriminations about sufficient evidence to satisfy a high criterion would likely magnify biases. Use of a liberal criterion would not solve the problems of biases, but would at least allow women authors to get their potentially important findings and ideas into the literature.

## Joachim Vandekerckhove

Throughout this discussion, we have often reflected on the status of the scientific literature. There certainly appears to be a duality, at least, in how the literature is perceived. If one thinks of it as mostly a vehicle for reporting the careful low-level observations that researchers have made, then there is no reason to limit what observations can be published as long as authors can be trusted to be truthful in their communiqués. If, on the other hand, one believes that the scientific literature serves mostly to promulgate robust claims about natural laws and regularities, then studies that provide only small amounts of evidence should perhaps not clutter the field.

In my experience, the literature is all of these things: It contains reports of clever studies that individually contribute bits of evidence; there are brilliant ideas about the workings of nature or how to carve it; and I occasionally see high-level integrations of larger bodies of work that lay significant questions to rest.

As Morey points out, it’s very unfortunate that our current publication ecosystem is such a hybrid beast that does not clearly differentiate between these functions of the scientific literature. Some of the questions we have asked here might be more easily answered if these different goals of publication were made more explicit and salient—I would say a “synthesis” literature could set certain standards that an “observations” or “ideas” literature does not need.

Circling back to the more recent topic of biases: If such a system came to exist, publishers could be in a position to design more customized policies to combat the various biases that have been discussed. For example, a literature with selective publication criteria could focus on blind review more, while another could encourage “light touch” peer review. One could encourage preregistration, while another could target a broad readership, and so on.

## Rich Shiffrin

The diverse viewpoints expressed in this dialogue illustrate some of the many complexities that arise when producing science. Is there a “best” method? This discussion shows that the criterion that ought to be used for publishing seemingly unlikely results is a matter for debate. This issue is likely not unique: Many other aspects of the way we practice science are unclear. We believe this dialogue has been useful and interesting, and similar dialogues concerning other practices could also be illuminating.
